# Electromagnetic informed data model considerations for near-field DOA and range estimates

**DOI:** 10.1038/s41598-024-65644-7

**Published:** 2024-07-03

**Authors:** Zohreh Ebadi, Amir Masoud Molaei, Muhammad Ali Babar Abbasi, Simon Cotton, Anvar Tukmanov, Okan Yurduseven

**Affiliations:** 1 Institute of Electronics, Communications, and Information Technology, Queen’s University Belfast, BT3 9DT Belfast, UK; 2 BT Labs, Adastral Park, BT Group, Ipswich, UK

**Keywords:** Electrical and electronic engineering, Information theory and computation

## Abstract

Localizing sources in the near-field is one of the emerging challenges for array signal processing, which has received a great deal of attention in recent years. The development of accurate localization algorithms requires the definition of a reliable model of the received signal that takes into account all wavefront characteristics, such as angle, range, and polarization, as well as electromagnetic effects, such as mutual coupling between antennas and the amplitude and phase behaviour of electromagnetic wavefronts. A system model that considers the electromagnetic-informed wave behaviour effects, independent of the type of receiver antennas, array structure, degree of correlation of sources signals and other electromagnetic effects, is considered an *“ exact model ”* in the literature. However, due to the mathematical complexity of this modeling approach, simplifications using several approximations are conventionally used. For instance, the phase of the exact model is approximated using the Fresnel approximation, while the magnitude of the exact model is simplified by assuming equal distances between the source and all elements in the array. In this work, we evaluate the accuracy of a localization algorithm, the multiple signal classification (MUSIC), using the exact and approximated models in the near-field region. Through a series of simulations, we demonstrate that the localization algorithm designed based on the electromagnetic-informed exact model outperforms the one designed using the approximated model. We also show that considering electromagnetic factors in the system model through the exact model results in a 13% improvement in the direction of arrival (DOA) root mean square error (RMSE) and a 57.7% improvement in range RMSE at signal-to-noise ratio (SNR) of 15 dB.

## Introduction

Localizing signal sources finds many applications in wireless communications, navigation, radar, sonar, etc.^1,2^. In this context, localization algorithms, based on the distance between the source and the antenna array, can be developed in two areas, the near-field and far-field^3,4^. If the source is in the far-field, i.e., Fraunhofer region, the electromagnetic wavefront can be modeled as a planar wave^5^. Therefore, the received wave spatially characterizes only the direction of arrival (DOA) of the wave. In contrast, if the source is not within the Fraunhofer region, the electromagnetic wavefront exhibits a spherical-wave behaviour^5^. Hence, in the near-field, both DOA and range information can be extracted^6,7^. In this case, because the source is in the near-field (i.e., Fresnel region), the conventional far-field localization methods such as those presented in^8–10^ need further considerations in an attempt to localize the source in the near-field.

Over the last few decades, numerous algorithms, such as those presented in^2,6,11–29^, have been proposed to estimate the DOA and range of the source in the near-field region. These algorithms rely on modeling the wavefront. In the near-field, the effects due to electromagnetic phenomena, such as mutual coupling between antennas and the radiation field of the receiver and transmitter, must be considered to model the received signal accurately. However, to the best of our knowledge, most near-field localization methods do not consider critical effects that are not prominent in the near-field on the signal model. Instead, they use a so-called *“ exact model ”* that is actually based on the behaviour of far-field electromagnetic waves^16,17,30,31^. The phase of the far-field electromagnetic wave is proportional to the product of the distance and inverse of the wavelength^32^. This phase differs from the phase of electromagnetic waves in the near-field^31^. Furthermore, the magnitude of the exact model is proportional to the inverse of the distance, which is valid when the source is in the far-field. However, the magnitude of the exact model ignores crucial terms e.g., the inverse of the square of the distance.

A limited number of works, such as^7,16,17,30^, have highlighted algorithms based on the exact model. However, these algorithms are only useful for particular antenna configurations, such as bistatic multiple-input multiple-output (MIMO) structure, and are unsuitable for other antenna arrays. Methods such as multiple signal classification (MUSIC) and maximum likelihood^10^, which rely on a search performed on defined grid points, can be used for the exact model. However, these methods can become computationally intensive as the number of unknowns increases - such as when the elevation angle, azimuth angle, and range are all unknown. This is because they require searching through multidimensional grid points.

Many methods for near-field localization, such as those described in^2,11–13,23–29^, rely on a simplified version of the exact model. In the near-field, unlike the far-field, the distance between the source and each element in the receiver array cannot be neglected. Therefore, a square root appears in the exact model phase based on the distance definition. Hence, the first simplification of the exact model is simplifying its phase term to a second-order Taylor polynomial known as the Fresnel approximation^33^. The Fresnel approximation will cause errors in the localization of the source in real scenarios^3,31,33^. The second simplification is ignoring the amplitude factor in the exact model. This simplification is only accurate when the source is located at a far distance from the array^31^. This assumption is no longer valid in the near-field, where the distance difference between the source and the array elements cannot be neglected, particularly for large arrays such as massive MIMO systems^32^. This simplified version of the exact model is referred to as the *“ approximated model ”* in this work.

In this work, we show the importance of taking near-field factors into account (rather than neglecting them), which are mostly ignored in the development of near-field localization algorithms. This way, we show the importance of considering the near-field effects and the fact that whereas the assumptions of the approximated model might be sufficient in the far-field, in the near-field, they produce highly inaccurate results. Hence, in the near-field, we investigate the accuracy of assumptions made in creating the approximated model^17^. We employ the MUSIC algorithm^8^, which is suitable for any array structure, to estimate the range and DOA using both exact and approximated models. In this research, we use two types of received signals: first is those generated by MATLAB using the exact model, and second is signal generated using a full wave electromagnetic simulation software, namely CST Microwave Studio^34^. Since the methods such as those described in^2,11–13,28,29^ utilize the signal generated based on the approximated model, the accuracy of these methods needs to be examined for the signals generated based on the exact model or actual signals measured through an experiment.

The main contributions of this paper are listed as follows:

- We investigate the validity of the assumptions that are considered for deriving the exact and approximated models.
- Using an electromagnetic full-wave simulation, we examine the compatibility of the exact and approximated models with electromagnetic-informed actual signals.
- We determine the accuracy of near-field localization methods for the signals generated using the exact model.

The significance of this work is to demonstrate the importance of considering electromagnetic-informed wave behaviour when analysing DOA and range estimation methods, independent of the specific type of antennas that the receiver might have. As a result, the type of array and the mutual coupling between its elements are not the main concern of this paper.

## System model

**Figure 1:**
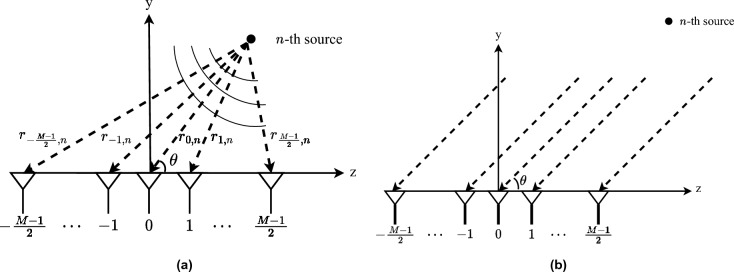
A simple representation of the system model; (**a**) in the near-field, (**b**) in the far-field.

Consider *N* narrowband signals impinging on a uniform linear array (ULA) with *M* elements, where *M* is an odd number. The inter-element spacing of elements is $d={\lambda }/{2}$, where $\lambda$ is the signal wavelength. The signal at the output of the ULA can be expressed as^13^

1 $$\begin{aligned} {\textbf{y}}_l = {\textbf{A}}(\mathbf {\theta },{\textbf{r}}){\textbf{s}}_l+{\textbf{w}}_l, \end{aligned}$$

where ${\textbf{s}}_l=[s_1(l)\ s_2(l)\ \cdots \ s_N(l)]^T$ is the vector of the transmitted non-coherent signals, ${\textbf{w}}_l=[w_1(l)\ w_2(l)\ \cdots \ w_M(l)]^T$ is white Gaussian noise with a zero mean and variance $\sigma ^2$, and $l=1,2,\cdots ,L$. *L* is the number of the snapshots. Furthermore, $\varvec{\theta }=[\theta _{1}\ \theta _{2}\ \cdots \ \theta _{N}]$ is the DOA vector of the received signals at the reference element, i.e., zeroth element in the ULA, and ${\textbf{r}}=[r_{0,1}\ r_{0,2}\ \cdots \ r_{0,N}]$ is the vector of range of sources where the range is defined as the distance between the *n*-th source and the reference element, as depicted in Fig. 1. Besides, ${\textbf{A}}(\varvec{\theta },{\textbf{r}})=[{\textbf{a}}(\theta _1,r_{0,1})\ {\textbf{a}}(\theta _2,r_{0,2})\ \cdots \ {\textbf{a}}(\theta _N,r_{0,N})]$ is $M\times N$ steering matrix. ${\textbf{a}}(\theta _n,r_{0,n})$ is $M\times 1$ steering vector, where each element in the steering vector is given by^31^

2 $$\begin{aligned} {\textbf{a}}(\theta _{n},r_{0,n}) = \frac{r_{0,n}}{r_{m,n}}e^{-j\tau _{m,n}}, \end{aligned}$$

where $\tau _{m,n}$ is the phase difference between the *n*-th source and *m*-th element in the array as follows:

3 $$\begin{aligned} \tau _{m,n}= \frac{2\pi }{\lambda }(r_{m,n}-r_{0,n}), \end{aligned}$$

where $r_{m,n}$ is the distance between the *n*-th source and the array’s *m*-th element. For the sake of simplicity and without loss of generality, we assume that the source and the ULA are in the horizontal plane (yz-plane in Fig. 1) and $m=-\frac{M-1}{2},\ \cdots ,\ -1,\ 0, \ 1,\ \cdots ,\ \frac{M-1}{2}$. Therefore, $r_{m,n}$ is given by^31^

4 $$\begin{aligned} r_{m,n} = \sqrt{r^2_{0,n}+m^2d^2-2mdr_{0,n}\cos {\theta _n}}~. \end{aligned}$$

We refer to (2) as the exact model. The common approach in the literature to estimate the DOA and range of the source relies on the simplification of (2) by considering some assumptions as detailed in the subsequent sections.

### Near-field case

If the source is in the near-field (Fresnel) region of the antenna array, $r_{0,n}\in (0.62(D^3/\lambda )^\frac{1}{2},2D^2/\lambda )$^6,12^, then the spherical wavefront of the source can not be approximated as a planar wave at the receiver, as shown in Fig. 1a. In this depiction, $D=(M-1)d$ is the aperture size of the ULA.

To properly describe the spherical wavefront, it is important to take into account both the DOA and the range. Within this region, the distance difference between the source and each element in the receiver array cannot be neglected. For instance, $r_{0,n}$ cannot be considered equal to $r_{1,n}$. However, a considerable amount of work in the literature assumes that $\frac{r_{0,n}}{r_{m,n}}\approx 1$ in the near-field^2,13,31^.

Furthermore, to simplify (4), the Fresnel approximation is used as^12^

5 $$\begin{aligned} \begin{aligned} r_{m,n} \approx r_{0,n}[1+\frac{m^2d^2}{2r^2_{0,n}}-\frac{md}{r_{0,n}}\cos {\theta _n}-\frac{m^2d^2\cos ^2{\theta _n}}{2r^2_{0,n}}] \\ \approx r_{0,n}+\frac{m^2d^2}{2r_{0,n}}\sin ^2{\theta _n}-md\cos {\theta _n}. \end{aligned} \end{aligned}$$

Therefore, $\tau _{m,n}$ can be written as

6 $$\begin{aligned} \tau _{m,n}\approx \gamma _nm+\eta _n m^2, \end{aligned}$$

where

7 $$\begin{aligned} \begin{aligned} \gamma _n=-\frac{2\pi d}{\lambda }\cos {\theta _n}, \\ \eta _n=\frac{\pi d^2}{\lambda r_{0,n}}\sin ^2{\theta _n}. \end{aligned} \end{aligned}$$

Based on the above-mentioned assumption and Fresnel approximation, (2) is simplified as follows:

8 $$\begin{aligned} {\textbf{a}}(\theta _{n},r_{0,n}) = e^{j(\gamma _nm+\eta _n m^2)}. \end{aligned}$$

We refer to (8) as the *approximated* model.

### Far-field case

Suppose the source is sufficiently far from the antenna array, specifically in the far-field (Fraunhofer) region, where the distance between the source and the array is much greater than the array aperture ($r_{0,n}>> D$)^31^. In that case, the spherical wavefront of the source can be approximated as a plane wave when it reaches the receiver (see Fig. 1b).

The DOA is the sole determinant of this plane wave, and its range is not a factor in its characterization in this region since it is considered infinite^20^. Therefore, to simplify the magnitude of the exact model, the approximation of $\frac{r_{0,n}}{r_{m,n}}\approx 1$ is used. Moreover, in the far-field, as $r_{0,n}>> md$, the term $(md)^2$ can be neglected with respect to $r^2_{m,n}$ in (4). Therefore, the remaining terms in the phase of the exact model in (4) can be simplified as:

9 $$\begin{aligned} r_{m,n} \approx r_{0,n}-md\cos {\theta _n}~. \end{aligned}$$

Therefore, (2) is simplified as

10 $$\begin{aligned} {\textbf{a}}(\theta _{n}) = e^{-j\frac{2\pi }{\lambda }md\cos {\theta _n}}~. \end{aligned}$$

## Validity of assumptions

In the following, we investigate the validity of assumptions considered to derive the exact and approximated models. These assumptions involve taking into account $\frac{r_{0,n}}{r_{m,n}}$ and $-\frac{2\pi }{\lambda }(r_{m,n}-r_{0,n})$ as magnitude and phase of the exact model, respectively. Therefore, this section examines whether the exact model commonly used in signal processing works is valid based on the electromagnetic theory. To do this, we investigate the phase and amplitude behaviour of the electromagnetic wave produced by the electric field and magnetic field.

In our analysis, we purposely consider a simple type of antenna, i.e., a dipole antenna, to highlight the impact of suboptimal assumptions on localization. Since the trend of the phase and magnitude of the electric and magnetic fields are similar, we only focus on the electric field^31^.

The electric field of a dipole antenna in the yz-plane can be expressed as follows^31^:

11 $$\begin{aligned} \begin{aligned} {\varvec{E}}=\frac{I\Delta z}{4\pi }[\frac{j\omega \mu }{r}+\sqrt{\frac{\mu }{\epsilon }}\frac{1}{r^2}+\frac{1}{j\omega \epsilon r^3}]e^{-j\frac{2\pi }{\lambda } r}\cos \theta \ {{\textbf {v}}} \\ +\frac{I\Delta z}{2\pi }[\sqrt{\frac{\mu }{\epsilon }}\frac{1}{r^2}+\frac{1}{j\omega \epsilon r^3}]e^{-j\frac{2\pi }{\lambda } r}\sin \theta \ {{\textbf {u}}}, \end{aligned} \end{aligned}$$

where *r*, *I*, $\Delta z$, $\omega$, $\mu$, and $\epsilon$ are the distance from the dipole, current in the dipole, dipole length, frequency, permeability, and permittivity of the propagation medium, respectively. Moreover, ${{\textbf {v}}}=[0,-\sin \theta ,\cos \theta ]^T$ and ${{\textbf {u}}}=[0,\cos \theta ,\sin \theta ]^T$ are unit direction vectors.

Based on (11), it is evident that the electric field amplitude decreases as the range increases. Therefore, in the far-field region, the terms including $\frac{1}{r^2}$ and $\frac{1}{r^3}$ are negligible. Hence, in the far-field region, (11) can be simplified as

12 $$\begin{aligned} {\varvec{E}}=\frac{jI\Delta z\omega \mu }{4\pi r}e^{-j\frac{2\pi }{\lambda } r}\cos \theta \ {{\textbf {v}}}. \end{aligned}$$

By comparing (11) and (12), it can be concluded that the phase of the electric field in the far-field, $-\frac{2\pi }{\lambda }r$, is different from the phase of the electric field in the near-field. Because the phase of ${\varvec{E}}$ in the near-field region is $-\frac{2\pi }{\lambda }r+\phi$, where $-\frac{2\pi }{\lambda }r$ is the phase of $e^{-j\frac{2\pi }{\lambda }r}$. Besides, $\phi$ includes $\frac{\pi }{2}$ for the term with *j* in the numerator and $-\frac{\pi }{2}$ for the term with *j* in the denominator. Therefore, the difference between the phase of the electric field in the far-field and near-field region is $\phi$. Besides, the phase of the electric field in the far-field region is exactly equal to the phase of the received signal by the center element in the antenna array, $-\frac{2\pi }{\lambda } r_{0,n}$, which is modelled using exact model, i.e., (2). In other words, the exact model and its approximation are based on the far-field electric field model.

In sequel, using computer simulations, we will demonstrate how these models lead to distinct errors in both DOA and range estimation.

## Results and discussion

In this section, we first examine the validity of assumptions introduced in the Validity of Assumptions Section with a full wave simulation. Next, we conduct electromagnetic simulations to evaluate the DOA and range estimation accuracy using both the exact and approximated models.

### Investigation of assumptions

**Figure 2:**
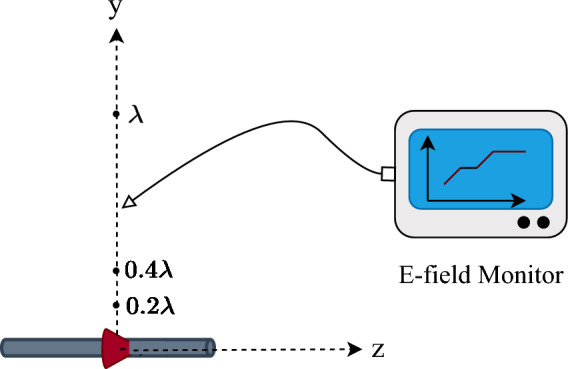
Dipole simulation setup in CST to measure the magnitude and phase of the dipole electric field.

Consider a half-wavelength dipole antenna operating at a frequency of 5 GHz (see Fig. 2). This simulation measures the electric field generated by the dipole at different distances along the y-axis.

**Figure 3:**
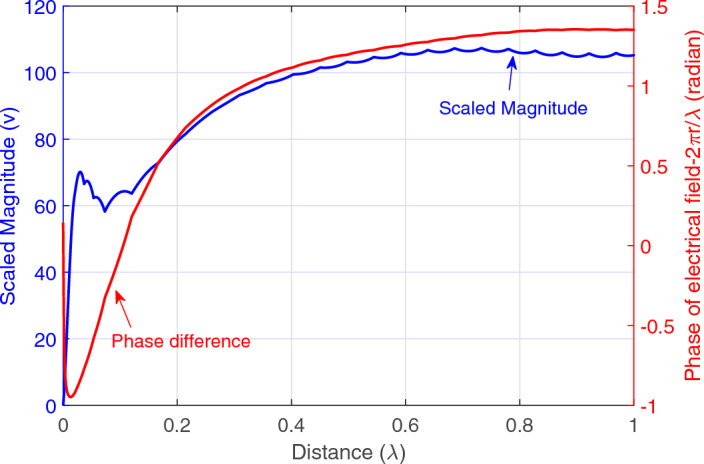
Scaled magnitude of the electric field, and subtraction of electric field phase and $2\pi r/\lambda$.

The magnitude of the exact model is only proportional to $\frac{1}{r}$, as expressed in (2). Therefore, the scaled magnitude, which is the product of distance and electric field (i.e., $r{\varvec{E}}$) ^31^, should be constant and independent of distance. To examine whether $r{\varvec{E}}$ is independent of distance, for one dipole antenna, the scaled magnitude of the electric field is plotted in Fig. 3 (blue graph). Hence, Fig. 3 shows that the magnitude of the electric field in the near-field of the antenna (distances less than $0.5\lambda$) is not only proportional to $\frac{1}{r}$ and also includes the other terms as expressed in (11). This confirms that the magnitude in the exact model includes only the $\frac{1}{r}$ term and neglects other terms. This magnitude in the approximated model is considered as 1.

Figure 3 (red graph) plots the subtraction of the phase of the electric field and $\frac{2\pi }{\lambda }r$ to show the difference of the phase in the near-field and far-field^31^. As Fig. 3 shows, the phase of the electric field in the near-field differs from $\frac{2\pi }{\lambda }r$. However, this fact is not considered in the exact and approximated models.

### Root mean square error (RMSE) of unknowns

In this subsection, the transmitted signal ${\textbf{s}}_l$ in (1) is a Gaussian random process^35^ with zero-mean, which is generated by MATLAB. The received signal ${\textbf{y}}_l$ in (1) is generated using the exact model i.e., (2). The simulation is carried out under the condition where the DOA and range of a single source are $[\theta _1,r_{0,1}]$. The receiver is a ULA consisting of seven elements with a frequency of 5 GHz in a sub-6 GHz frequency band. Therefore, in the near-field, $r_{0,1}$ can be in the range of $(3.2\lambda ,18\lambda )$. To generalize our work, we conducted simulations for DOAs ranging from $20^\circ$ to $70^\circ$ in increments of $10^\circ$. The number of snapshots is 200. All simulation results are obtained based on the average of $K=1000$ independent trials.

We use the MUSIC algorithm to estimate unknowns. This algorithm relies on the eigen decomposition of the covariance matrix of received signal (1) to extract the signal and noise subspaces (${{\textbf {U}}}_s$ and ${{\textbf {U}}}_w$). Then, the unknowns can be provided by searching for peaks of the MUSIC spectrum which is as follows^7^

13 $$\begin{aligned} P(\theta ,r)=\frac{1}{{{\textbf {a}}}^H{{\textbf {U}}}_w{{\textbf {U}}}_w^H{{\textbf {a}}}}. \end{aligned}$$

where each element in $\textbf {a}$ is defined using (2) and (8) for the MUSIC algorithm based on the exact model (MUSIC-EM) and approximated model (MUSIC-AM), respectively. RMSE^12^ of the DOA and range are used to compare the exact with the approximated

14 $$\begin{aligned} {\textrm{RMSE}}_\alpha =\sqrt{\frac{1}{KN}\sum _{k=1}^{K}\sum _{n=1}^{N}({\hat{\alpha }}_{n,k}-\alpha _{n,k})^2}. \end{aligned}$$

where $\alpha$ and ${\hat{\alpha }}$ represent the true value and the estimated value of DOA or range, respectively.

**Figure 4:**
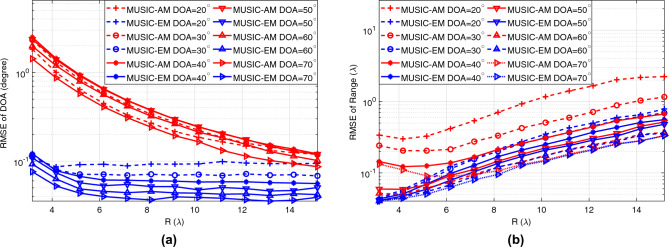
RMSE of estimated (**a**) DOA, (**b**) range versus distance. *R* represents different values of $r_{0,1}$.

In the first simulation, the signal-to-noise ratio (SNR), defined as the ratio of signal power to noise power at the receiver, is 15 dB. Here, the term signal refers to ${\textbf{y}}_l$ in (1). Figure 4 plots the RMSE of the estimated DOA and range versus different range values. The figures show that the MUSIC-EM outperforms the MUSIC-AM for all ranges and DOAs. This is due to the fact that the signal is generated based on the exact model. Hence, the MUSIC-EM is compatible with the data generated by the exact model. In Fig. 4a, it can be observed that the RMSE of the DOA estimated through MUSIC-EM is consistently less than $0.13^\circ$ for all ranges within the Fresnel region. Moreover, as the range increases, the RMSE of the DOA estimated through MUSIC-AM tends to converge towards the RMSE of the DOA estimated through MUSIC-EM. As shown in Fig. 4b, the RMSE of the estimated range using MUSIC-AM and MUSIC-EM increases with the range.

**Figure 5:**
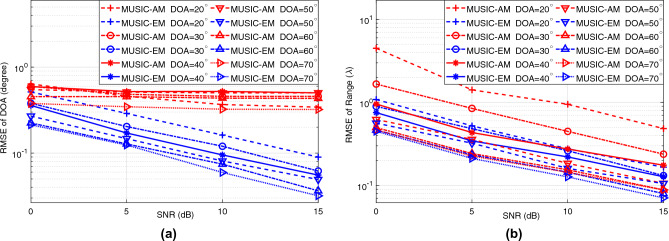
RMSE of estimated (**a**) DOA, (**b**) range versus SNR.

Following the investigation of DOA and range estimation RMSEs for the MUSIC-EM and MUSIC-AM models under a constant SNR level (15 dB) in Fig. 4, we study the impact of the SNR on the estimator’s performance for both models. For this study, the range is chosen to be $7\lambda$. As shown in Fig. 5, the RMSE of estimated unknowns using the MUSIC-EM is less than the RMSE of those estimated by the MUSIC-AM for all DOA and SNR values. Figure 5 also shows that the RMSE of the estimated unknowns using MUSIC-EM and MUSIC-AM decreases as SNR increases.

These simulation results show that, for the given simulation setup in this section, if the localization method is developed based on the exact data model, they can localize the source more accurately than methods based on the approximated model for all ranges, DOAs, and SNRs.

### Data generated using full-wave electromagnetic solver

**Figure 6:**
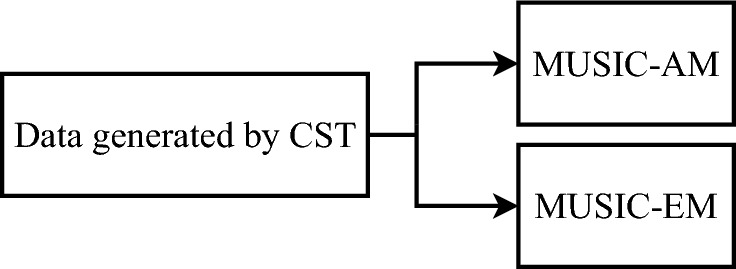
Data generated by CST studio suite and processed by MUSIC-EM and MUSIC-AM.

In this subsection, we conduct a full-wave electromagnetic simulation using CST Microwave Studio^34^ to validate the results provided in the previous subsection. The full-wave CST simulations take into account all electromagnetic-related factors, and, therefore, the generated dataset will be fully electromagnetic-informed. Following from this, we use the MUSIC-EM and the MUSIC-AM to estimate unknowns, as shown in Fig. 6.

All simulation parameters are the same as those in RMSE of Unknowns subsection. In this simulation, the receiver is a ULA consisting of seven dipole antennas. The source is a single dipole. We conducted simulations for various DOA values, including $50^\circ$ and $70^\circ$.

**Figure 7:**
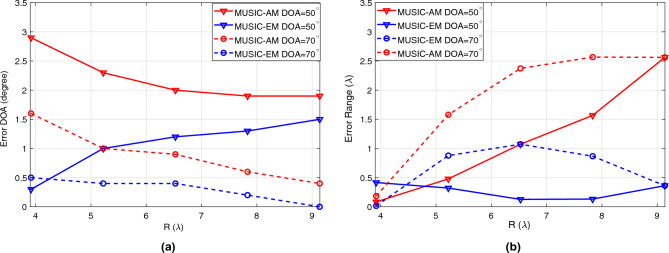
Error of estimated (**a**) DOA, (**b**) range versus distance. *R* represents different values of $r_{0,1}$.

According to Fig. 7a, the error of estimating the DOA using the MUSIC-EM is consistently lower than using the MUSIC-AM across all ranges, where the error is defined as $|{{\hat{\alpha }}}-\alpha |$. Moreover, in Fig. 7b, it can be seen that in most cases, the estimation error for the range is lower when using the MUSIC-EM compared to the MUSIC-AM. This is due to the fact that the implementation of the algorithm based on the exact model (compared to the approximated model) is more consistent with the actual signal generated by CST. These results prove that developing algorithms based on the exact signal model can significantly reduce the estimation error in real-world scenarios.

It should be noted that although the results presented in Figs. 4 and 7 are self-consistent, comparing Figs. 4, 5, 6, 7, distinct behaviours are observed due to the different methods used to generate their results. The results shown in Fig. 7 were obtained through a full-wave electromagnetic simulation. Therefore, these results also take into account several additional factors, such as the mutual coupling between the components in the receiver array and the directive beam patterns of the dipole antennas forming the array. However, as discussed earlier, the exact and approximated models are concerned with the near-field and far-field electromagnetic behaviour of the source generated waves rather than the mutual coupling and beam shape. To produce the results shown in Fig. 4, it can be considered that the signal is generated using a calibrated array (consisting of omnidirectional elements), i.e., without mutual coupling. Therefore, Figs. 4 and 7 are not expected to be the same. The purpose of generating the received signal using the full-wave simulation was to demonstrate that, even for an uncalibrated antenna array with directive beam patterns, using a system model that closely resembles the realistic model will result in less estimation error than the approximated model.

### The impact of the system model on the accuracy of estimating unknowns

In the previous subsections, we demonstrated that the MUSIC-EM provides more accurate estimates for unknowns compared to the MUSIC-AM. In addition to MUSIC-EM and MUSIC-AM, we consider methods based on the approximation models developed in^2,13,28,29^ in this subsection. Methods in^13,29^ need an even number of elements in the ULA, while methods in^2,28^ need an odd number of elements. *M* for methods in^2,13,29^ and^28^ are considered to be 6 and 7, respectively. Moreover, *d* in method^13^ is $\lambda /2$ while *d* in methods^2,28,29^ is less than or equal to $\lambda /4$. The reason is that in the method^13^, DOA is estimated using the steering vector $e^{jm\gamma _n}$, while in the methods^2,28^ and^29^, the DOA is estimated using the steering vector $e^{j2m\gamma _n}$^13^.

We use these methods to estimate unknowns for two types of received signals: those generated based on the approximated model and those generated based on the exact model. In this simulation, the SNR and the number of snapshots are 10 dB and 200, respectively. Since the interelement spacing *d* for different methods are different, we chose the location of the source such that it falls within the Fresnel region for each method. Hence, $r_{0,n}$ for method in^2,13,28^, and^29^ is $1.6\lambda$, $5\lambda$, $1.16\lambda$, and $1.3\lambda$, respectively. Furthermore, $r_{0,n}$ for MUSIC-AM and MUSIC-EM is $7\lambda$. Moreover, the DOA of source is $50^\circ$. Table 1 indicates that all methods, except the MUSIC-EM method, result in lower RMSE for signals generated with the approximated model compared to the exact model. This is because the methods are developed assuming an approximated model, making them more compatible with it than the exact model. As a result, these methods will not work well in practical scenarios because they are only reliable for signals simulated using the approximate model. In contrast, methods developed using the exact model of the received signal, such as the MUSIC-EM method, work accurately.

**Table 1:** The impact of the data model on the accuracy of estimating unknowns.

Method	Exact model		Approximated model	
	DOA RMSE	Range RMSE	DOA RMSE	Range RMSE
^2^	$2.15^\circ$	$0.03\,\!\lambda$	$0.09^\circ$	$0.02\,\!\lambda$
^13^	$0.96^\circ$	$0.56\,\!\lambda$	$0.3^\circ$	$0.4\,\!\lambda$
^28^	$2.34^\circ$	$0.003\,\!\lambda$	$0.2^\circ$	$0.002\,\!\lambda$
^29^	$1.77^\circ$	$1.15\,\!\lambda$	$0.57^\circ$	$1.02\,\!\lambda$
MUSIC-AM	$0.5^\circ$	$0.18\,\!\lambda$	$0.07^\circ$	$0.19\,\!\lambda$
MUSIC-EM	$0.08^\circ$	$0.16\lambda$	$0.3^\circ$	$1.19\lambda$

## Conclusion

The first step in designing a localization algorithm in the near-field and far-field regions is creating a proper system model that includes electromagnetic effects and signal characteristics. The system model that takes into account electromagnetic effects is referred to as the exact model in the near-field. The exact model can be simplified considering several assumptions, and it is important to assess the validity of these assumptions to gain a comprehensive understanding of the localization problem.

We first examined the derivation of the exact model and the assumptions considered to simplify it. The results showed that since the magnitude and phase of the electric field in the near-field and far-field are different, the assumptions are not accurate and need to be modified. Then, we generated signals based on the exact model and applied the MUSIC-EM and MUSIC-AM. The simulation results showed that the estimator’s accuracy depends on the system model used to develop that estimator. For instance, the MUSIC-AM cannot estimate the unknowns as accurately as the MUSIC-EM. We did a full-wave electromagnetic simulation to validate this result to generate a more realistic signal. Again, the accuracy of MUSIC-EM was higher than that of the MUSIC-AM. Additionally, we evaluated the performance of four localization techniques on signals generated using both the exact and approximated models. The accuracy of these methods significantly degrades when estimating DOA and the range of received signal modeled by exact model, as they are developed based on the approximated model. Our studies confirm that it is essential to use an electromagnetic-informed wireless system model to develop the localization method to enhance localization accuracy, especially in near-field regions.

## Data Availability

The datasets used and/or analysed during the current study available from the corresponding author on reasonable request.

## References

[1] Krim H, Viberg M (1996). Two decades of array signal processing research: The parametric approach. IEEE Signal Process. Mag..

[2] Zhang X, Chen W, Zheng W, Xia Z, Wang Y (2018). Localization of near-field sources: A reduced-dimension MUSIC algorithm. IEEE Commun. Lett..

[3] Zhang Q, Li W, Yang B, Li S (2023). An auxiliary source-based near field source localization method with sensor position error. Signal Process..

[4] Zhang W, Han Y, Jin M, Li X-S (2020). An improved ESPRIT-like algorithm for coherent signals DOA estimation. IEEE Commun. Lett..

[5] Balanis CA (2016). Antenna Theory: Analysis and Design.

[6] Zuo W (2019). Localization of near-field sources based on linear prediction and oblique projection operator. IEEE Trans. Signal Process..

[7] Huang Y-D, Barkat M (1991). Near-field multiple source localization by passive sensor array. IEEE Trans. Antennas Propag..

[8] Schmidt R (1986). Multiple emitter location and signal parameter estimation. IEEE Trans. Antennas Propag..

[9] Roy R, Kailath T (1989). ESPRIT-estimation of signal parameters via rotational invariance techniques. IEEE Trans. Acoust. Speech Signal Process..

[10] Stoica P, Sharman K (1990). Maximum likelihood methods for direction-of-arrival estimation. IEEE Trans. Acoust. Speech Signal Process..

[11] Liang J, Liu D (2010). Passive localization of mixed near-field and far-field sources using two-stage MUSIC algorithm. IEEE Trans. Signal Process..

[12] Molaei AM, del Hougne P, Fusco V, Yurduseven O (2022). Efficient joint estimation of DOA, range and reflectivity in near-field by using mixed-order statistics and a symmetric MIMO array. IEEE Trans. Veh. Technol..

[13] Li J (2021). DOA and range estimation using a uniform linear antenna array without a priori knowledge of the source number. IEEE Trans. Antennas Propag..

[14] Sheng, M., Xin, J., Zuo, W. & Zheng, N. Sparse Bayesian learning for near-field narrowband source localization. In *2020 Chinese Automation Congress (CAC)*, (IEEE, 2020). 10.1109/cac51589.2020.9326910

[15] Qiu L, Lan T, Wang Y (2019). A sparse perspective for direction-of-arrival estimation under strong near-field interference environment. Sensors.

[16] Singh P, Wang Y, Chargé P (2017). An exact model-based method for near-field sources localization with bistatic MIMO system. Sensors.

[17] Chen H, Wang W, Liu W, Tian Y, Wang G (2023). An exact near-field model based localization for bistatic MIMO radar with COLD arrays. IEEE Trans. Veh. Technology..

[18] He J, Swamy MNS, Ahmad MO (2012). Efficient application of MUSIC algorithm under the coexistence of far-field and near-field sources. IEEE Trans. Signal Process..

[19] Lu F (2022). Investigation of near-field source localization using uniform rectangular array. Electronics.

[20] Cheng C, Liu S, Wu H, Zhang Y (2023). Mixed-field source localization based on robust matrix propagator and reduced-degree polynomial rooting. Signal Process..

[21] Molaei AM, Zakeri B, Andargoli SMH (2019). Passive localization and classification of mixed near-field and far-field sources based on high-order differencing algorithm. Signal Process..

[22] Xue D (2023). Three-dimensional near-field localization with cross array considering amplitude attenuation. Circuits Syst. Signal Process..

[23] Cheng C, Liu S, Wu H, Zhang Y (2022). An efficient maximum-likelihood-like algorithm for near-field coherent source localization. IEEE Trans. Antennas Propag..

[24] Guanghui C, Xiaoping Z, Shuang J, Anning Y, Qi L (2020). High accuracy near-field localization algorithm at low SNR using fourth-order cumulant. IEEE Commun. Lett..

[25] Su X (2019). An SOS-based algorithm for localization of multiple near-field sources using uniform circular array. IEEE Sens. Lett..

[26] Lee J-H, Chen Y-M, Yeh C-C (1995). A covariance approximation method for near-field direction-finding using a uniform linear array. IEEE Trans. Signal Process..

[27] Grosicki E, Abed-Meraim K, Hua Y (2005). A weighted linear prediction method for near-field source localization. IEEE Trans. Signal Process..

[28] Zhi W, Chia MY-W (2007). Near-field source localization via symmetric subarrays. IEEE Signal Process. Lett..

[29] Li, J., Wei, G., Ma, B., Wang, Y. & Bastard, C. L. A simple way for near-field source localization with MUSIC. In *2016 IEEE International Conference on Computational Electromagnetics (ICCEM)*. 10.1109/compem.2016.7588682 (IEEE, 2016).

[30] Singh, P. R., Wang, Y. & Charge, P. Near field targets localization using bistatic MIMO system with spherical wavefront based model. In *2017 25th European Signal Processing Conference (EUSIPCO)*. 10.23919/eusipco.2017.8081642 (IEEE, 2017).

[31] Friedlander B (2019). Localization of signals in the near-field of an antenna array. IEEE Trans. Signal Process..

[32] Cui, M., Wu, Z., Lu, Y., Wei, X. & Dai, L. Near-field communications for 6G: Fundamentals, challenges, potentials, and future directions. *IEEE Commun. Mag.* **61**, 40–46. 10.48550/ARXIV.2203.16318 (2022).

[33] He J, Li L, Shu T, Truong T-K (2021). Mixed near-field and far-field source localization based on exact spatial propagation geometry. IEEE Trans. Veh. Technol..

[34] Studio, C. M. *CST Microwave studio* (CST Studio Suite, 2008).

[35] Zuo W, Xin J, Zheng N, Sano A (2018). Subspace-based localization of far-field and near-field signals without eigendecomposition. IEEE Trans. Signal Process..

